# Comparative Evaluation of Spreadability Measurement Methods for Topical Semisolid Formulations/A Scoping Review

**DOI:** 10.3390/gels11121006

**Published:** 2025-12-12

**Authors:** Elham Y. Al-Barghouthy, Saja Hamed, Ghadeer F. Mehyar, Hatim S. AlKhatib

**Affiliations:** 1 Department of Pharmaceutics & Pharmaceutical Technology, School of Pharmacy, The University of Jordan, Amman 11942, Jordan; alh9210022@ju.edu.jo; 2 Department of Pharmaceutics & Pharmaceutical Technology, Faculty of Pharmaceutical Sciences, The Hashemite University, Zarqa 13133, Jordan; hamedsh@hu.edu.jo; 3 Department of Nutrition and Food Technology, The University of Jordan, Queen Rania Street, Amman 11942, Jordan; g.mehyar@ju.edu.jo

**Keywords:** spreadability, texture analyzer, frictiometer, rheology, stress yield, semisolid formulations, tribology, standardization

## Abstract

Background: Spreadability is a critical performance attribute for semisolid formulations, influencing patient compliance, dose uniformity, and product acceptability. Despite its importance, there is no standardized method for its assessment across pharmaceutical and cosmetic applications. Objective: This review uniquely integrates systematic literature mapping with an experimental comparison of five spreadability assessment techniques, providing evidence-based recommendations for harmonizing protocols and improving reproducibility in semisolid formulation testing. Methods: A systematic search of PubMed, Scopus, and Web of Science identified 211 records, of which 14 studies met the inclusion criteria. Techniques reviewed included parallel-plate, slip-and-drag, rheometry (flow curve and amplitude sweep), texture analysis, and frictiometry. An experimental comparison was conducted on ten commercial formulations using all five techniques to assess inter-method variability and formulation-dependent behavior. Results: Texture analyzer and amplitude sweep rheometry emerged as the most reproducible and predictive methods, showing strong correlation (r = 0.74) in both literature and experimental data. Flow curve yield stress negatively correlated with parallel-plate spreadability (r = −0.796). Frictiometry results varied significantly with formulation type, particularly for ointments. Creams consistently ranked highest in spreadability across methods. Conclusion: No single method universally captures spreadability. Amplitude sweep rheometry correlated well with texture analysis, while flow curve values were more variable. Parallel-plate testing showed strong agreement with rheological and tribological methods, though texture analysis diverged, capturing distinct mechanical attributes. A tiered approach integrating parallel-plate, amplitude sweep, and frictiometry is recommended, with flow curve retained for regulatory compliance. Texture analysis provides valuable orthogonal information. Standardization of parallel-plate protocols is needed to establish unified spreadability indices.

## 1. Introduction

In the pharmaceutical and cosmetic industries, products must possess the correct physical and chemical properties as well as be acceptable to patients and consumers. Spreadability is a crucial factor in the consumer’s assessment of a topical product, as it has a direct impact on both the user experience and the product’s effectiveness [1]. A formulation with superior spreadability enables consistent dispersion of active substances, improves absorption, and increases patient compliance [2].

Spreading is a substance’s ability to cover a surface, which is determined by its molecular weight, viscosity, and chemical structure [3,4]. Spreadability is measured using a variety of methodologies, including subjective assessments and advanced instrumental procedures [5]. Spreadability is a key parameter in the structural (Q3) characterization of topical formulations, and both the United States Food and Drug Administration (FDA) and the European Medicines Agency (EMA) increasingly emphasize the use of standardized, instrument-based methods to assess it. Rheological profiling is explicitly referenced in regulatory guidance as a primary tool for evaluating the microstructural behavior of semisolid products [6]. Texture analyzers are also accepted [7]. In contrast, the parallel-plate method, despite its historical use in early-stage formulation screening, is not recognized by either the FDA or EMA as a standardized or complementary method for regulatory submissions. It lacks defined protocols for sample mass, applied weight, duration, and calculation equations, and its manual nature introduces variability that undermines reproducibility.

In this review, the methods used in measuring spreadability are classified in three major categories: subjective (sensory), physical (parallel-plate and slip-and-drag), and measuring instruments (rheological, texture analyzer, and Frictiometer).

### 1.1. Subjective Assessment (Sensory Assessment)

Subjective assessment of spreadability relies on volunteers’ tactile evaluation, offering realistic but non-standardized results [5]. To improve consistency, the International Organization for Standardization ISO (2003), established sensory analysis guidelines enabling both qualitative and quantitative evaluation of cosmetic features [8,9]. The standard guide for two sensory descriptive analysis approaches for skin creams and lotions (ASTM, 2011) outlines two key approaches: Technical Expert and Consumer Behavior [10]. Gore et al. (2018) trained expert assessors per ISO 8586-1 [11], using a 0–7 scale to rate application force [12]. Savary et al. (2013) quantified spreadability by measuring the distance a 50 μL emulsion could be moved on the skin [2].

Despite its precision, sensory testing is costly and resource-intensive, limiting its feasibility for smaller institutions and academic settings [13,14].

### 1.2. Physical Assessment

#### 1.2.1. The Parallel-Plate Method

The most common approach for determining spreadability is the parallel-plate method, which has numerous versions. It involves sandwiching a sample between two glass plates and applying a known weight on the upper plate. Several studies have employed diverse methods to assess semisolid spreadability. Inoue et al. (2017) used a Rigo spread meter to calculate the yield stress (Y_1_) of vidarabine creams based on plate weight, sample volume, and final spread diameter [15]. Neamah and Al-Akkam (2024) evaluated vemurafenib microemulsion in hydrogel by measuring the change in border diameter, calculating spreadability as a function of applied weight, migration distance, and time [16]. Deuschle et al. (2015) expressed the spreadability of calendula-based formulations as area per gram under applied weight [17]. The parallel-plate method, introduced by Keller et al. (1982) [18], remains a simple and cost-effective approach, though its reproducibility is limited by variations in sample mass, plate weight, time, and calculation methods [19]. Due to inconsistent parameters and load ranges (typically 100–500 g), results across studies using this method are often non-comparable. A summary of the equations cited in previous studies is presented in Table 1.

#### 1.2.2. Slip-and-Drag Method

The slip-and-drag method, a variation in the parallel-plate approach, involves placing a sample between two glass plates, applying weight, and measuring the time required for plate separation after removing the load [5]. Spreadability is calculated based on plate mass, slip distance, and separation time. Dubey et al. assessed a graphene nanoconjugate ointment using angled plates, recording the time for the upper plate to disengage after sliding 7.5 cm [20].

### 1.3. Measuring Instruments

#### 1.3.1. Rheometry or Viscometry (Yield Stress)

Rheology is the study of material flow (viscometry test, i.e., yield stress) and deformation (oscillatory test, i.e., strain/stress amplitude sweep and frequency sweep), and it is essential for understanding the spreadability of cosmetic and skincare products such as creams, gels, and emulsions [21,22,23]. Yield stress is a significant parameter linked to spreadability; it is inversely correlated to spreadability [5].

Yield stress can be evaluated using a rheometer, typically through flow curve analysis, by mathematical curve-fitting models [24] or amplitude sweep testing, at the end of the linear viscoelastic region (LVR) [13,25].

#### 1.3.2. Texture Analyzers

Texture analyzers offer objective, reproducible methods for evaluating spreadability by measuring the force required to spread a substance [26]. They also assess parameters like hardness, stiffness, and work of shear, making them valuable in food, pharmaceutical, and cosmetic industries [27,28]. Spreadability is typically measured using a conical probe system, where the flow between probe and container surfaces is quantified in gram·second (g·s), with peak force indicating firmness [29]. Studies have compared texture analyzer data with sensory and rheological assessments, showing strong correlations [30,31,32,33,34,35,36,37,38]. Gilbert et al. (2013) demonstrated that instrumental measurements, including viscosity and stiffness, effectively predict sensory attributes such as gloss, compression force, and spreadability (R^2^ > 0.96), confirming texture analyzers as reliable tools for sensory texture evaluation [39].

#### 1.3.3. Frictiometer

The Frictiometer FR 700 (Courage+Khazaka electronic GmbH, Cologne, Germany) quantifies skin friction using a motorized Teflon probe (Courage+Khazaka electronic GmbH, Cologne, Germany), with torque readings reflecting product slipperiness, spreadability, and stickiness [15]. Skin tribology has gained attention for its role in evaluating cosmetic interactions with human skin [40,41,42,43,44,45]. Studies like Gore et al. (2018) examined how oil phase composition affects emulsion spreadability, comparing sensory and instrumental data [12].

**Table 1:** Equations that are used with physical and instrumental methods for spreadability and spreadability-related parameters calculations (S: spreadability; Y: yield stress).

Method	Equation No.	Equations	Variables and Units	References
The parallel-plate	Equation (1)	$S_{1}=d^{2}\times \frac{\pi }{4}$	*S*_1_: Spreadability (mm^2^) and *d*: diameter of spread (mm).	[18]
	Equation (2)	$S_{2}=\frac{d^{2}\times \frac{\pi }{4}}{W}$	*S*_2_: Spreadability (mm^2^/g); *d*: diameter (mm); and *W*: weight applied on the sample (g).	[17]
	Equation (3)	$S_{3}=\frac{W\times d}{T\;}\;$	*S*_3_: Spreadability (g·mm/s), *W*: weight applied on the sample (g), *T*: time (s), and *d*: diameter (mm).	[16]
	Equation (4)	$Y_{1}=47040\times G\times \frac{V}{\pi^{2}\times D^{5}}$	*Y*_1_: Yield stress (Pa); *G*: gravitational constant; *V*: volume (cm^3^); and *D*: diameter (cm).	[15]
Slip-and-drag	Equation (5)	$S_{4}=W\times \frac{L}{t}$	*S*_4_: Spreadability parameter obtained from slip-and-drag (g·cm/s), with *W*: weight applied on the sample (g); the glass plate length, *L*: the distance that the upper plate had to slip for separation; and *t*: time (s).	[5,20]
Spreadability from Yield stress	Equation (6)	$S_{5}=\frac{1}{Y_{2}}$	*S*_5_: Spreadability parameter obtained from flow curve yield stress (Pa^−1^). *Y*_2_: yield point from flow curve (Pa)	[5]
Flow Curve Bingham	Equation (7)	*τ* = *Y*_2_ + *η*_*B*_·$\dot{\gamma }$	*τ*: Shear stress (Pa), *η*_*B*_: viscosity (Pa·s), and $\dot{\gamma }$: shear rate (s^−1^).	[24]
Flow Curve Casson	Equation (8)	√τ = *Y*_2_ + √(*η_c_*·$\dot{\gamma }$)	*τ*: Shear stress (Pa), *Y*_2_: yield point (Pa), *η_c_*·: viscosity (Pa·s), and $\dot{\gamma }$: shear rate (s^−1^).	[24]
Flow Curve Herschel–Bulkley	Equation (9)	*τ* = *Y*_2_ + *c*·$\dot{\gamma }$*^p^*	*τ*: Shear stress (Pa), *τ*: Herschel–Bulkley yield point (Pa), *c*: flow coefficient, $\dot{\gamma }$: shear rate (s^−1^), *p* < 1: shear-thinning, *p* > 1: shear-thickening, and *p* = 1: Bingham flow behavior.	[24]
Amplitude Sweep	Equation (10)	$S_{6}=\frac{1}{Y_{3}}$	*S*_6_: Spreadability parameter obtained from the yield stress obtained from the amplitude sweep (Pa^−1^) and *Y*_3_: the end of the LVR yield stress value.	[46]
Texture analyzer	Equation (11)	$S_{7}=\frac{1}{HW}$	*S*_7_: Spreadability parameter obtained from *HW*: Hardness work (mJ) and area under the positive curve of penetration force vs. time.	[29]
Frictiometer	Equation (12)	$S_{8}=\frac{F1\;}{F2}$	*S*_8_: Spreadability parameter obtained from (AU), *F*1: Baseline friction, and *F*2: friction after treatment (AU).	Embedded

With evolving analytical technologies, it has become essential to reassess how spreadability is measured in semisolid formulations. Techniques such as parallel-plate setups, slip-and-drag methods, rheometry, texture analysis, and frictiometry are widely used, yet they differ in sensitivity, reproducibility, and real-world relevance. Despite regulatory emphasis by the FDA and EMA on formulation-specific product performance, no standardized protocol currently exists. This study aims to systematically map and compare these instrumental methods across pharmaceutical and cosmetic applications. Through a scoping review and experimental evaluation of ten commercial products, we identify methodological trends, assess variability across techniques, and propose a practical, regulation-aware framework to guide formulation development and quality control.

## 2. Results and Discussion

### 2.1. Overview of Included Studies

A total of 14 studies were included in the scoping review, spanning publications from 1972 to 2024. These studies evaluated spreadability using one or more of the following techniques: parallel-plate method, slip-and-drag method, rheometry (flow curve and amplitude sweep), texture analyzer, frictiometry, and sensory assessment. The formulations assessed included creams, gels, and ointments, with varying oil content, viscosity, and structural properties.

### 2.2. Spreadability Measurement Techniques in Literature

#### 2.2.1. The Parallel-Plate Method Results

Nine studies employed the parallel-plate method, using various equations to calculate spreadability or yield stress. Units varied across studies (e.g., mm^2^, dyne/cm^2^, g·cm/s), complicating direct comparison. Spreadability was generally inversely correlated with yield stress, and results were sensitive to plate weight, sample volume, and time duration.

#### 2.2.2. The Slip-and-Drag Method Results

Four studies used slip-and-drag techniques, often as a variation in the parallel-plate method. Spreadability was calculated based on the time required for plate separation under a defined load. This method was less commonly standardized and showed moderate reproducibility.

#### 2.2.3. Rheometry Results

Eight studies assessed yield stress using rheometers. Flow curve analysis (via Herschel–Bulkley, Bingham, or Casson models) and amplitude sweep tests were used to determine the yield point. Amplitude sweep yield stress showed a stronger correlation with sensory and texture analyzer data than flow curve-derived values.

#### 2.2.4. The Texture Analyzer Results

Six studies used texture analyzers to measure spreadability via penetration force and work of shear. Results were expressed in gram·second (g·s) and showed high reproducibility. Texture analyzer data correlated well with sensory attributes such as firmness and ease of spreading.

#### 2.2.5. The Frictiometry Results

Three studies employed frictiometers to measure skin friction during product application. Friction values were inversely related to spreadability and positively associated with stickiness. Results varied significantly with formulation type, especially for high-oil-content ointments.

#### 2.2.6. Sensory Assessment

Several studies incorporated trained panels or consumer evaluations to assess tactile spreadability. While valuable for realism, sensory methods were time-consuming, costly, and subject to inter-panel variability.

Cyriac et al. (2023) demonstrated that instrumental rheological, tribological, and textural parameters can be effectively correlated with sensory attributes using Principal Component Analysis (PCA) [47]. Their study showed that spreadability is positively correlated with material parameters obtained from Large-Amplitude Oscillatory Shear (LAOS) responding to large deformation rheology rather than small deformation rheology. This includes parameters like minimum rate, dynamic viscosity, and large-strain elastic shear modulus. On the other hand, spreadability showed no correlation with linear rheological parameters or parameters from steady shear measurements.

**Figure 1:**
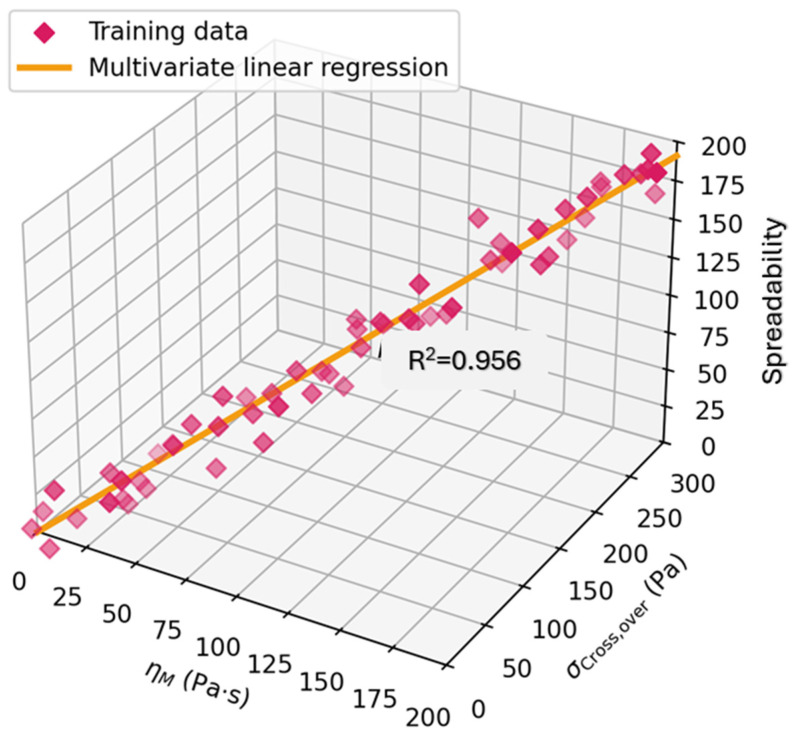
Multivariate linear regression model showing the predictive relationship between minimum dynamic viscosity (η_m_), cross-over stress (σ_cross over_), and spreadability (N·s) of topical formulations. Magenta diamonds represent training data, and the orange regression line demonstrates a strong fit, with R^2^ = 0.95612. The model illustrates how rheological parameters can be used to forecast spreadability, supporting data-driven formulation design. Adapted from Cyriac et al. (2023) [47].

Figure 1 demonstrates a multivariate linear regression model showing the predictive relationship between minimum dynamic viscosity (η_m_), cross-over stress (σ_cross over), and spreadability (N·s) of topical formulations. Magenta diamonds represent training data, and the orange regression line demonstrates a strong fit, with R^2^ = 0.95612. The model illustrates how rheological parameters can be used to forecast spreadability [47].

### 2.3. Correlation Trends Across Methods

Texture analyzer vs. sensory: Strong correlation (R^2^ > 0.9) in three studies.

Amplitude sweep yield stress vs. texture analyzer: Positive correlation (r = 0.73–0.75).

Flow curve yield stress vs. parallel-plate: Negative correlation (r = −0.796).

Frictiometer vs. texture analyzer: Significant differences observed (*p* < 0.05), especially for ointments.

### 2.4. Embedded Case Study: Experimental Comparison

To validate and contextualize the literature findings, an experimental comparison was conducted using ten commercial semisolid formulations (three creams, three gels, three ointments, and soft paraffin). Each product was evaluated using five techniques: the parallel-plate method (four equations), the slip-and-drag method, rheometry (flow curve and amplitude sweep), the CTX texture analyzer (Brookfield Ametek, Middleboro, MA, USA), and the Frictiometer (Courage-Khazaka Electronic GmbH, Germany). All results are summarized in Table 2 and Table 3.

#### 2.4.1. Statistical Overview

##### Pearson Correlation

**Figure 2:**
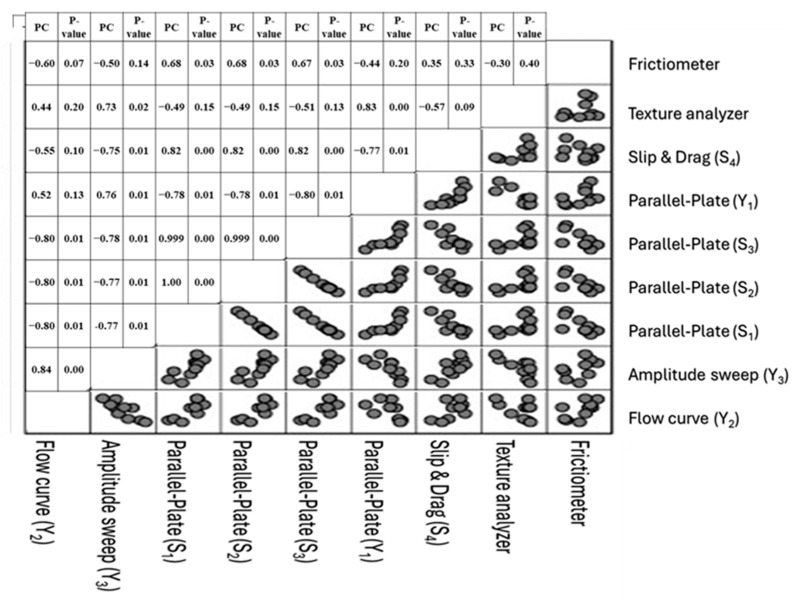
Pearson correlation scatterplot matrix for the parameters used to evaluate spreadability and mechanical behavior of semisolid formulations. Scatter plots illustrate pairwise relationships among frictiometry, texture analysis, slip-and-drag (S_4_), parallel-plate parameters (Y_1_, S_1_–S_3_), flow curve yield stress (Y_2_), and amplitude sweep (Y_3_), highlighting degrees of agreement and divergence across methods. PC: Pearson correlation. *p*-value is the two-tailed *p*-value.

To assess the strength and direction of the linear relationship between the spreadability parameters obtained using different methodologies, Pearson correlation analysis was performed. Pearson’s correlation coefficient analysis (Figure 2) indicated the following:

Flow curve yield stress vs. parallel-plate spreadability (Equation (1) in Table 1):

(r = −0.796); (*p* = 0.006) → strong negative correlation.

Amplitude sweep yield stress vs. texture analyzer, slip-and-drag, and parallel-plate (Equation (2) in Table 1):

(r = 0.732–0.748); (*p* < 0.016) → strong positive correlation.

##### Repeated ANOVA Correlation

**Figure 3:**
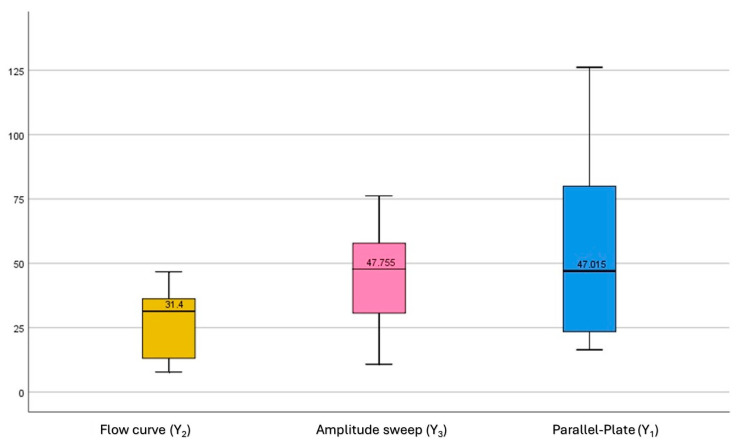
Analysis of box plots illustrating comparison of yield stress values obtained from three rheological methods: flow curve (Y_2_), amplitude sweep (Y_3_), and parallel-plate (Y_1_). Box plots illustrate median values, interquartile ranges, and data variability, with amplitude sweep and parallel-plate methods showing higher central tendencies and broader distributions than flow curve measurements.

The yield values inversely correlate with spreadability (i.e., higher yield stress implies lower spreadability). The analysis of the boxplots and median yield, shown in Figure 3, reveals no significant outliers. Yield stress values determined via the parallel-plate method (Y_1_) demonstrated strong statistical harmony with those derived from the amplitude sweep test (Y_3_), as indicated by a non-significant *p*-value of 0.775 (*p* ≥ 0.05).

##### Friedman Test

Significant differences in spreadability rankings across methods:

(*p* < 0.05) for comparisons involving the texture analyzer vs. the frictiometer, parallel-plate, and flow curve.

#### 2.4.2. Method-Specific Observations

**Figure 4:**
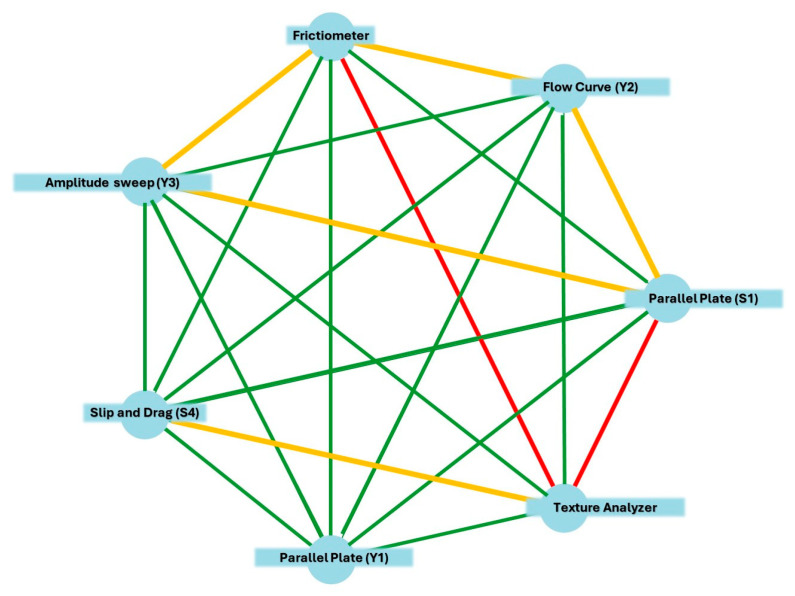
Network plot of Friedman ranking tests for pairwise comparisons between spreadability measurement methods. Each node represents a method, and edges represent pairwise comparisons based on Bonferroni-adjusted significance values. Red dashed edges indicate statistically significant differences, yellow double-lined edges indicate statistically moderately significant differences, and green solid edges indicate non-significant comparisons.

Friedman’s pairwise comparisons between the different spreadability measurement methods revealed both statistically significant and non-significant differences, as visualized in Figure 4. The Bonferroni-adjusted pairwise tests identified two method pairs with statistically significant ranking differences at the α = 0.05 level: the texture analyzer differed significantly from both the frictiometer (adjusted *p*-value = 0.006) and the parallel-plate (S_1_) method (adjusted *p*-value = 0.006). These findings suggest that the texture analyzer captures spreadability characteristics in a manner distinct from the other instruments tested.

#### 2.4.3. Technique Key Findings

Parallel-plate: Results varied by equation; Equation (1) in Table 1 (area-based) showed highest sensitivity to formulation type. The yield stress (Y_1_) results obtained from Equation (4) in Table 1 correlated with the ranking results of the other six methods.

Slip-and-drag: This method demonstrated moderate reproducibility; gels showed faster plate separation than ointments.

Rheometry amplitude: Sweep yield stress aligned well with that of the texture analyzer; flow curve values were more variable. These findings are consistent with those of Cyriac et al. (2023), who reported that non-linear rheological parameters from amplitude sweep (LAOS) aligned more robustly with texture analyzer outputs, while flow curve-derived values exhibited greater variability and model fitting challenges [47].

Texture analyzer: This method produced distinct spreadability values compared to most others and correlated best with amplitude sweep.

Frictiometer: This method was sensitive to residual film and oil content; ointments showed higher friction and lower spreadability.

#### 2.4.4. Formulation Ranking Trends

Creams consistently ranked highest in spreadability across most methods.

Gels showed method-dependent variability:

They ranked high in slip-and-drag and frictiometer methods, while ranking lower in texture analyzer and amplitude sweep methods.

Ointments ranked lowest overall, especially with the texture analyzer and frictiometer, due to high adhesiveness and friction.

#### 2.4.5. Summary of Findings

This scoping review identified five principal techniques used for spreadability assessment in semisolids: parallel-plate, slip-and-drag, rheometry, texture analysis, and frictiometry. Across the 14 included studies, texture analysis and amplitude sweep rheometry emerged as the most reproducible and predictive, particularly when linked to sensory performance.

The embedded experimental study supported these findings, showing a strong correlation between amplitude sweep yield stress and texture analyzer outcomes (r = 0.74). Importantly, parallel-plate yield stress (Y_1_) produced ranking results that aligned closely with all other methods, suggesting that—despite lacking formal standardization—this technique may serve as a practical, low-cost alternative for routine screening when used under controlled conditions.

#### 2.4.6. Methodological Insights

The literature revealed substantial variability in how spreadability is defined and measured. Parallel-plate results differed depending on the equation used; slip-and-drag lacked standardized parameters; flow curve rheometry was sensitive to model fitting; texture analysis offered consistent mechanical profiles; and frictiometry provided realistic skin-contact data but showed high variability, particularly with oily formulations.

In the embedded study, parallel-plate yield stress calculated using Equation (4) (Y_1_) produced ranking patterns statistically comparable to six other methods. Similarly, the slip-and-drag technique showed non-significant pairwise differences with five instrumental methods (adjusted *p* = 0.807–1.000), indicating consistent rank distribution. These results highlight that simple, low-resource methods can approximate the performance of advanced instruments.

However, broader application of manual approaches remains limited by the absence of standardized protocols. Variability in sample mass, applied force, timing, and equation selection reduces reproducibility across laboratories. Standardization of these techniques is essential to enhance reliability and support potential integration into regulatory Q3 characterization frameworks recognized by the FDA and EMA. Establishing validated procedures would improve scientific rigor while expanding access to spreadability assessment in resource-limited environments.

#### 2.4.7. Practical Implications for Formulation Science

For formulators and cosmetic scientists, the results suggest a tiered approach to spreadability assessment:

- Primary screening using a texture analyzer and amplitude sweep rheometry for reproducibility and mechanical insight;
- Secondary validation using frictiometry or sensory panels to capture user experience and skin interaction;
- Avoid sole reliance on parallel-plate or slip-and-drag methods unless standardized equations and conditions are used.

This approach balances analytical rigor with practical relevance, especially in regulatory or consumer-facing contexts.

#### 2.4.8. Limitations

This review was limited to English-language publications and excluded sensory-only studies without instrumental data. The embedded case study focused on ten commercial formulations, which may not represent the full diversity of semisolid products. Additionally, inter-operator variability and equipment calibration were not assessed.

#### 2.4.9. Future Directions

Future research should aim to

- Develop a unified spreadability index that integrates mechanical, rheological, and sensory dimensions;
- Standardize test conditions, equations, and units across methods;
- Explore machine learning models to predict sensory outcomes from instrumental data;
- Expand frictiometry protocols to account for skin type, hydration, and application dynamics and correlate specific genetic markers and ethnicity with variations in skin function and response;
- Align each formulation type and its intended application with the most appropriate method for spreadability assessment.

## 3. Conclusions

This scoping review mapped the landscape of spreadability measurement techniques for semisolid formulations, identifying five core methods with varying reproducibility and relevance. Texture analysis and amplitude sweep rheometry emerged as the most robust and predictive techniques, while parallel-plate and frictiometry showed formulation-dependent variability.

The embedded experimental study validated these trends, highlighting the texture analyzer as a method producing distinct spreadability values compared to most others.

Parallel-plate (Y_1_) ranking results demonstrated strong correlation with the outcomes of all other methods. This highlights the potential of the parallel-plate approach as a cost-effective tool for spreadability assessment, owing to its simplicity, affordability, and ease of use in routine formulation screening. Although not yet formally standardized, its ranking performance showed close alignment with more advanced techniques, supporting its practical utility under controlled conditions. Establishing clear standards for the applied weight, measurement time, sample amount, and calculation method would enhance its consistency and reliability, positioning it as a convenient and economical option for spreadability evaluation alongside flow curve analysis.

Our findings support a tiered evaluation framework that combines parallel-plate mechanical testing, amplitude sweep rheometry, and frictiometry as complementary techniques for robust spreadability assessment. While flow curve analysis remains mandated under United States Food and Drug Administration (FDA) Q3 characterization, its variability underscores the importance of additional methods to ensure reproducibility. Texture analysis, though distinct from rheological and tribological measures, contributes valuable orthogonal insights into firmness and adhesiveness that enrich application-focused evaluation. Harmonizing these protocols and developing unified spreadability indices that integrate rheological, mechanical, and tribological data will strengthen both regulatory compliance and practical formulation design.

## 4. Materials and Methods

Ten over-the-counter topical preparations currently available on the market were selected for the study. Three creams, three gels, and three ointments: Barrad gel (HiGeen/Amman/Jordan), Doloraz gel (JPM/Amman/Jordan), PremaRil AR gel (Premium Innovation/Jordan), Betaval cream (Noripharma/Klang/Malaysia), Clipp cream (Clippcare/Beirut/Lebanon), Harrar cream (HiGeen/Amman/Jordan), Betaval ointment (Noripharma/Klang/Malaysia), Dermovate ointment (Taropharma, Brampton, Ontario/Canada), Nayyar ointment (Skin Horizons/Amman/Jordan), and soft paraffin (Sana Pharma/Amman/Jordan). The composition of each product alongside its pharmacological indications is listed in Appendix A Table A1.

### 4.1. Experimental Procedures

#### 4.1.1. Parallel-Plate Method

A 2 cm^3^ of the topical preparation was placed between two circular glass plates of an in-house parallel-plate apparatus, as shown in the Supplementary Figure S1. The upper glass plate, weighing 1057.09 g, was lowered on top of the sample, and the sample’s diameter was then measured after 1 min. Five consecutive measurements were taken at room temperature (20–25 °C), with a new sample placed for each measurement, and the spreadability was calculated for each measurement. Spreadability (S) by the parallel-plate method was calculated using the following Equations (1)–(4). Then, the average and the standard deviation were calculated for each set of calculations

#### 4.1.2. The “Slip-and-Drag” Method

A 1 cm^3^ topical preparation was placed on a glass plate attached to a wooden block of an “in-house” slip-and-drag apparatus at RT (20–25 °C). The sample was then covered with another glass plate for 30 s to ensure the removal of air bubbles. A weight block (50.0 ± 0.1 g) was placed on a holder attached to the upper plate at a 90° angle using a pulley [19]. A graphical illustration is shown in Supplementary Figure S2. The time for the upper plate to completely separate from the lower plate was measured in seconds. Three consecutive measurements were taken, with a new sample placed for each measurement, and the spreadability was calculated for each one. The spreadability (S) and standard deviation were then determined for each topical preparation using Equation (5).

#### 4.1.3. Rheological Method

To obtain the rheological measurements of the products, a rotational rheometer (Physica, MCR 301, Anton Paar GmbH, Graz, Austria) was used with a stainless-steel cone-plate geometry with a 50 mm diameter, at a constant temperature of 25 ± 0.2 °C, and a gap size of 0.051 mm. Flow curve and strain amplitude sweep were determined for each topical preparation. The flow curve was measured within a stress range of 0.001 Pa to 10,000 Pa at a ramp time of 2 min and a decade of 10. The Herschel–Bulkley model was used for yield stress calculation [48]. Data was analyzed by GraphPad Prism 8.4.3.686 software.

For the strain amplitude sweep, the samples were oscillated over a shear strain range of 0.01% to 100%, at a frequency of 75 s^−1^. This rheological test, where the strain (or deformation) applied is varied while the frequency is kept constant, helps determine the linear viscoelastic region (LVR), which is crucial for understanding how the material behaves under stress. The measuring results are presented as a diagram, with strain plotted on the x-axis and storage modulus G′ and loss modulus G″ plotted on the y-axis on a logarithmic scale, from which the limit of the linear viscoelastic region (LVR), as well as the yield stress, is calculated.

As shown in Supplementary Figure S3, GraphPad Prism 8.4.3.686 software was utilized to determine best-fit lines using a two-segment linear regression model and constraining the first slope to be around zero for data obtained from the rheometer, where Y: log G′ and X: log τ. At the end of the LVR, yield stress value (Y_3_) was obtained.

#### 4.1.4. Texture Analyzer

Using the CTX texture analyzer (Brookfield Ametek, Middleboro, MA, USA), a penetration test was carried out to assess the texture characteristics of the products, after being held at 25 °C for two to three hours. The instrument was outfitted with a male 45° cone probe and perfectly matching female Teflon cone-shaped product holders (Locally designed); Supplementary Figure S4. The sample was inserted into the female cone, and the male cone penetrated the sample to a depth of 47 mm at the rate of 3 mm/s. The textural data (force vs. time) were analyzed by the instrument software (TexturePro CT software V1.10 Brookfield Ametek, Middleboro, MA, USA). While the probe moves down, the product flows outward between the surfaces of the cone-shaped container and the probe. The area above zero represents the spreadability of the sample (see Supplementary Figure S5). The peak of the positive part of the graph shows the products’ ability to flow and their firmness. All measurements were taken in duplicate, and the findings are expressed as the mean ± SD.

Hardness (or firmness) is defined as the highest point on the positive curve, indicating the maximum work required to penetrate the formulation, measured in grams (g). Additionally, the work performedperformed in terms of hardness, measured in millijoules (mJ), is represented by the area under the positive curve, which reflects spreadability [26]. The test was performed in duplicates. The reciprocal of the hardness work done is the spreadability parameter.

#### 4.1.5. Frictiometer

A frictiometer equipped with a plain, smooth Teflon (PTFE) disk measuring 16 mm in diameter (Courage-Khazaka Electronic GmbH, Germany), was used to measure the friction coefficient of the topical products on the forearms of healthy human volunteers. The volunteers, who had no history of skin disease, were recruited from the university personnel at the Faculty of Pharmaceutical Sciences at Hashemite University and the University of Jordan. Ethical approval for the study was obtained from the Hashemite University Institutional Review Board, and informed consent was obtained from all volunteers (IRB No. 2500043/78). Fifteen female volunteers, aged 20 to 50 (Average 29.066 ± 7.38), participated in the frictiometer study. All participants had healthy skin at the measurement sites and refrained from using any skin care products in those areas for at least 2 h before testing. The study was conducted in a controlled environment at a temperature of 22 ± 1 °C and a relative humidity of 46 ± 4.8%, and they were allowed to acclimate for 20 min with bare forearms before measurement. On the volar side of each forearm, five square areas measuring 2.5 cm by 2.5 cm were designated for treatment. These areas were positioned both proximally and distally, ensuring a minimum spacing of 0.5 cm between neighboring sites and at least 3 cm between the proximal and distal ends. Volunteers were given 20 min to relax and adjust to the measurement setting, during which the test regions were exposed to the ambient air of the test environment.

Using a randomized design, 0.03 mL of each formulation was applied to each test site using a 1 mL syringe. Each application involved 10 circular motions performed by a latex-gloved finger, followed by 6 s of spreading using a frictiometer at a speed of 90 rpm. Immediately afterward, the frictiometer was used to measure the friction coefficient of the residual film on the skin using a rotation speed of 90 rpm for 20 s. The software provided the data in arbitrary units (A.U.). To minimize bias and ensure uniform film thickness on the skin, all applications were conducted by the same operator.

As a control, the friction value of bare skin before product application at each location was also recorded using a rotation speed of 90 rpm for 20 s, taking one measurement per second.

The results were expressed as the average ratio of baseline friction area (measured before treatment) to friction area measured immediately after treatment, calculated for 15 subjects for each formulation [44].

#### 4.1.6. Statistical Analysis

Spreadability parameters obtained from the abovementioned methodologies, S (1–4) in parallel-plate and slip-and-drag methods, the reciprocal of Y (1–3) in parallel-plate (Equation (4)) and rheometry (flow curve and amplitude sweep), and the reciprocal of the hardness work performed in the texture analyzer, or the frictiometer ratio in the frictiometer, were statistically analyzed using IBM SPSS version 26 for the ten products.

Pearson’s correlation coefficients were determined between different types of instrumental data to highlight significant correlations (*p*-value = 0.05) between them.

Friedman’s test, a nonparametric alternative to repeated-measures analysis of variance, compares observations from the same subject. This test was chosen to examine spreadability findings obtained through different methodologies because, like many nonparametric tests, Friedman’s test calculates statistics based on data ranks rather than raw values [49], which means that the unit or dimension of the reading is neglected. Moreover, unlike the parametric repeated-measures ANOVA, this test makes no assumptions regarding the normality of data distribution.

Friedman’s test was applied in two ways; the first test examined the consistency of formulation rankings across methods (whether different evaluation methods ranked the formulations’ spreadability in a similar way or showed significant differences), while the second test evaluated differences between methods in ranking the formulations (whether the different methods varied in how they ranked the formulations). Since the Friedman test ranks values from smallest to largest, direct comparisons between methods (second approach) could be influenced by differences in measurement scales rather than the actual product rank in each method. To standardize rankings across different measurement methods, a new variable called “relative spreadability” was introduced. This variable is based on the observation that in sensory tests, researchers typically identify soft paraffin (Petrolatum) as having poor spreadability [50]. So, relative spreadability can be identified as the ratio of formulation’s spreadability parameter using a certain measuring method to the soft paraffin spreadability parameter using the same method. By computing relative spreadability, we ensure that all results use the same scale, effectively removing discrepancies caused by variations in raw measurement values. This allows for a more consistent comparison of spreadability across methods.

Relative spreadability = (spreadability parameter of formulation)/(spreadability parameter of soft paraffin).

Since yield values obtained from rheological tests (flow curve and amplitude sweep), as well as from parallel-plate data (Equation (4)), are in the same units, repeated-measures ANOVA is useful for evaluating the obtained yield stresses. Since the same samples are tested using different methods, repeated-measures ANOVA is suitable for evaluating whether the yield values significantly differ across methods while accounting for within-sample variability.

### 4.2. Scoping Review Methodology

#### 4.2.1. Information Sources and Search Strategy

Databases: PubMed, Scopus, and Web of Science.

Search string: (“spreadability” OR “ease of spreading”) AND (“semisolid” OR “topical” OR “cosmetic”) AND (“texture analyzer” OR “rheometer” OR “frictiometer” OR “parallel plate” OR “slip and drag”). No date or language restrictions were applied. The search was conducted between 5 August and 20 August 2025.

#### 4.2.2. Eligibility Criteria

Included: studies evaluating the spreadability of semisolid formulations using physical/instrumental methods.

Excluded: oral/injectable formulations, sensory-only studies, and inaccessible full texts.

Study Selection and Screening.

**Figure 5:**
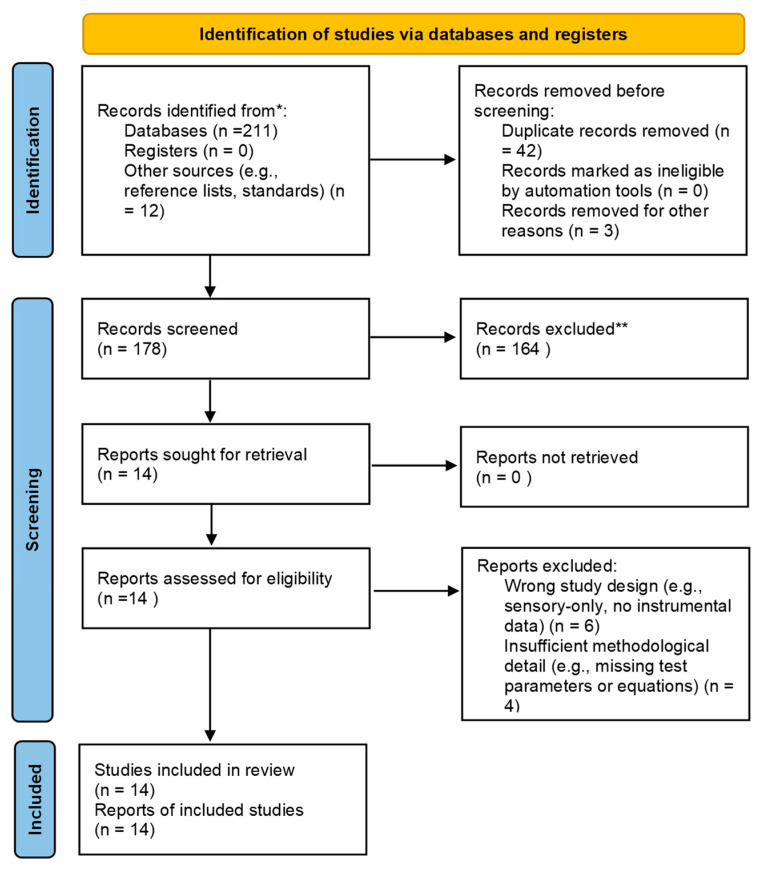
PRISMA-ScR flow diagram illustrating the identification, screening, and inclusion of studies assessing spreadability techniques for semisolid formulations. Records were identified through database searches (n = 211) and other sources such as reference lists (n = 12). * Databases searched included [PubMed, Scopus, and Web of Science]. After removing duplicates and irrelevant entries (n = 45), 178 records were screened. ** Records excluded during screening were removed based on title and abstract irrelevance to inclusion criteria; of these, 154 were excluded based on title and abstract. Twenty-four full-text reports were assessed for eligibility, with 10 excluded for the following reasons: six due to unsuitable study design (e.g., sensory-only with no instrumental data), and four due to insufficient methodological detail (e.g., missing test parameters or equations). Fourteen studies were included in the final review.

The study selection process followed a predefined protocol, which was registered on the Open Science Framework (OSF) (https://osf.io/zhsvm, 14 November 2025). Screening is performed in two stages: title/abstract, and then full-text. The PRISMA-ScR flow diagram summarizes this process (Figure 5).

#### 4.2.3. Data Charting and Synthesis

Extracted: method used, formulation type, spreadability metric, units, and correlation findings.

We categorized references as evidence-based vs. contextual.

#### 4.2.4. Embedded Case Study

Experimental Comparison of Spreadability Techniques

In addition to reviewing the existing literature, we conducted an experimental comparison of ten commercially available semisolid formulations, including creams, gels, and ointments. This comparison employed five different techniques for assessing spreadability: the parallel-plate method, the slip-and-drag test, rheometry (which includes flow and amplitude sweep), texture analysis, and frictiometry. The purpose of this case study was to illustrate the variability between these different methods and to validate the comparative insights drawn from the literature. The methodology and results of this embedded study are integrated throughout the review to provide context for the findings and to highlight their practical implications. For detailed experimental information, refer to Section 2.1.

## Figures and Tables

**Table 2:** Spreadability parameters assessed by manual physical assessment methods. Each cell shows the values, representing the mean ± standard deviation for each parameter, while (R) is the ranking score, with rankings from 1 to 10 based on the spreadability parameter of each column, where 1 indicates the lowest and 10 the highest spreadability. Parameters were obtained using the parallel-plate method (d, S_1_–S_3_, Y_1_) and slip-and-drag method (Time, S_4_).

Formulation	d (cm)		S_1_ (cm^2^)		S_2_ (mm^2^/g)		S_3_ (g·cm/s)		Y_1_ (Pa)		Time (s)		S_4_ (g·cm/s)	
	Value	R	Value	R	Value	R	Value	R	Value	R	Value	R	Value	R
**Barrad gel^®^**	3.94 ± 0.23	**6**	12.20 ± 1.42	**6**	1.15 ± 0.13	**5**	69.35 ± 4.03	**6**	47.31 ± 14.02	**5**	8.80 ± 1.54	**5**	104.2 ± 16.63	**5**
**Doloraz gel^®^**	3.83 ± 0.14	**2**	11.50 ± 0.85	**2**	1.09 ± 0.08	**2**	67.37 ± 2.45	**2**	46.72 ± 7.81	**6**	4.16 ± 0.85	**8**	221.70 ± 40.35	**8**
**PremaRil AR gel^®^**	4.12 ± 0.27	**7**	13.36 ± 1.70	**7**	1.26 ± 0.16	**7**	72.54 ± 4.78	**7**	33.08 ± 13.07	**7**	5.41 ± 0.54	**6**	167.3 ± 16.68	**6**
**Betaval cream^®^**	4.93 ± 0.40	**10**	19.18 ± 3.01	**10**	1.81 ± 0.28	**10**	86.83 ± 7.08	**10**	16.44 ± 8.23	**10**	2.60 ± 0.49	**10**	355.7 ± 74.94	**10**
**Clipp cream^®^**	4.75 ± 0.30	**9**	17.81 ± 2.23	**9**	1.69 ± 0.21	**9**	83.77 ± 5.22	**9**	23.50 ± 7.30	**8**	4.45 ± 0.33	**7**	203.2 ± 15.36	**7**
**Harrar cream^®^**	4.46 ± 0.19	**8**	15.63 ± 1.34	**8**	1.48 ± 0.13	**8**	78.54 ± 3.40	**8**	19.54 ± 4.38	**9**	4.04 ± 2.17	**9**	261.6 ± 108.77	**9**
**Betaval ointment^®^**	3.55 ± 0.15	**1**	9.94 ± 0.85	**1**	0.94 ± 0.08	**1**	62.62 ± 2.69	**1**	126.09 ± 27.55	**1**	11.52 ± 3.54	**2**	82.73 ± 22.46	**2**
**Dermovate ointment^®^**	3.82 ± 0.24	**3**	11.52 ± 1.47	**3**	1.09 ± 0.14	**3**	67.36 ± 4.25	**3**	99.72 ± 29.39	**2**	15.74 ± 3.39	**1**	59.05 ± 13.05	**1**
**Nayyar ointment^®^**	3.93 ± 0.10	**5**	12.12 ± 0.61	**5**	1.15 ± 0.06	**6**	69.20 ± 1.74	**5**	51.04 ± 6.35	**4**	9.95 ± 3.82	**3**	103.4 ± 50.57	**4**
**Soft paraffin**	3.86 ± 0.04	**4**	11.70 ± 0.26	**4**	1.11 ± 0.02	**4**	68.00 ± 0.76	**4**	79.98 ± 4.54	**3**	9.62 ± 3.50	**4**	100.9 ± 30.40	**3**

**Table 3:** Spreadability parameters assessed by measuring instruments methods. Each cell shows the values, representing the mean ± standard deviation for each parameter, while (R) is the ranking score, with rankings from 1 to 10 based on the spreadability parameter of each column, where 1 indicates the lowest and 10 the highest spreadability. Parameters were obtained using flow curve and amplitude sweep yield values (Y_2_, Y_3_), texture analyzer (hardness work), and frictiometer (ratio).

Formulation	Y_2_ (Pa)		Y_3_ (Pa)		Hardness Work (HW) (mJ)		Frictiometer Ratio (Baseline/Treatment)		Friedman’s Tests
	Value	R	Value	R	Value	R	Value	R	Mean Ranking
**Barrad gel^®^**	31.645	5	57.810	3	17.95 ± 0.35	5	0.95 ± 0.51	2	4.57
**Doloraz gel^®^**	45.464	2	56.364	4	13.10 ± 0.42	7	0.69 ± 0.41	1	4.14
**PremaRil AR gel^®^**	31.150	6	30.690	8	12.70 ± 1.70	8	1.08 ± 0.64	4	6.57
**Betaval cream^®^**	10.860	9	18.408	9	14.75 ± 0.49	6	2.05 ± 0.97	9	8.86
**Clipp cream^®^**	13.139	8	37.325	7	20.55 ± 2.19	4	1.97 ± 0.73	8	7.43
**Harrar cream^®^**	7.813	10	10.814	10	9.95 ± 0.07	10	1.44 ± 0.94	7	9
**Betaval ointment^®^**	36.232	3	64.269	2	57.80 ± 1.41	2	0.99 ± 0.44	3	2
**Dermovate ointment^®^**	21.719	7	52.360	5	39.15 ± 3.89	3	1.23 ± 0.76	6	3.71
**Nayyar ointment^®^**	32.082	4	43.152	6	10.60 ± 2.55	9	2.14 ± 1.60	10	6
**Soft paraffin**	46.701	1	76.208	1	63.00 ± 3.39	1	1.19 ± 0.54	5	2.71

## Data Availability

The review protocol was registered on the Open Science Framework (OSF) [https://osf.io/zhsvm, 14 November 2025]. Literature search data are available within the article and Supplementary Materials. Experimental datasets generated during the study are available from the corresponding author upon reasonable request.

## Supplementary Materials

The following supporting information can be downloaded at: https://www.mdpi.com/article/10.3390/gels11121006/s1, Figure S1: Parallel-plate schematic., Figure S2: Schematic of the “in-house” device used in the “slip-and-drag” method. Figure S3: Yield stress estimation using the method of De Graef et al. (2011) [46]: intersection of tangential lines of storage modulus at low and high oscillatory stresses. Figure S4: Schematic of the “in-house”-designed conical fixture used in the texture analyzer. Figure S5: Typical output graph obtained using the texture analyzer.

## Abbreviations

The following abbreviations are used in this manuscript:

AU	Arbitrary Unit
EMA	The European Medicines Agency
FDA	The United States Food and Drug Administration
LVR	Linear Viscoelastic Region
OSF	Open Science Framework
PTFE	equipped with a plain, smooth Teflon

## Appendix A

**Table A1:** Medicinal and non-medicinal ingredients of topical formulations obtained from the market with their pharmacological use.

Product Name	Manufacturer	Active Ingredient	Non-Medicinal Ingredients	Indication
Barrad Gel^®^	HiGeen, Jordan	Menthol	Aqua (Water), Isopropyl alcohol, Propylene glycol, Triethanolamine, Carbomer, Diazolidinyl urea, Methylparaben, Propylparaben	Relief from musculoskeletal pain by its anesthetic & counter-irritant properties
Doloraz Gel^®^	JPM, Jordan	Ibuprofen	Carbomer, Propylene Glycol, Diisopropanolamine, Ethanol, Purified Water	Anti-inflammatory and pain killer (NSAID)
PremaRil AR Gel^®^	Premium Innovation, Jordan	Horse chestnut extract Arnica extract	Water; Sodium Laureth Sulfate; Propylene Glycol; Distearyl Phthalamic Acid; Lauryl Glucoside; Aloe Vera Leaf; Diisodecyl Adipate; Glycerin; Hydrogenated Polybutene (1300 MW); Polyacrylamide (1500 MW); Cetostearyl Alcohol; Titanium Dioxide; Arnica Montana Flower; Green Tea Leaf; Cetyl Alcohol; Magnesium Aluminum Silicate; Polyoxyl 20 Cetostearyl Ether; Laureth-7; PEG-100 Stearate; C13-14 Isoparaffin; Levomenthol; Glyceryl Monostearate; Xanthan Gum; Methylparaben; Glutaral; Chlorphenesin	Fast recovery from bruising, swelling, and pain
Betaval Cream^®^	Noripharma, Malaysia	Betamethasone valerate	Water; Mineral Oil; Petrolatum; Lanolin Alcohols; Ceteth-20; Cetostearyl Alcohol; Sodium Phosphate, Monobasic, Anhydrous; Phosphoric Acid; Sodium Hydroxide; Chlorocresol	Topical corticosteroid for eczema, psoriasis, dermatitis
Clipp Cream^®^	Clippcare, Lebanon	None	Aqua (Water); Glycerin; Cetearyl Alcohol; Octyldodecanol; Beeswax; Caprylic/Capric Triglyceride; Glyceryl Stearate; Parfum (Fragrance); Sodium Cetearyl Sulfate; Phenoxyethanol; Allantoin; Dimethicone; Sorbitan Caprylate; Sodium Hydroxide; Citral; Geraniol; Linalool; Benzyl Benzoate; Citronellol; Limonene)	cosmetic moisturizer
Harrar Cream^®^	HiGeen, Jordan	Thyme, eucalyptus, turpentine and menthol	Aqua (Water), Stearic acid, Propylene glycol, Mineral oil, Glycerin, Cetearyl alcohol, Saccharide isomerate, Potassium hydroxide Ammonia, Acrylates/C10-30 alkyl acrylate crosspolymer, Diazolidinyl urea, Methylparaben, Propylparaben	Relieves pain arising from arthritis & tendonitis
Betaval Ointment^®^	Noripharma, Malaysia	Betamethasone valerate	White Soft Paraffin; Propylene Glycol; Liquid Paraffin; Glyceryl Monostearate; Methyl Parahydroxybenzoate	Topical corticosteroid for eczema, psoriasis, dermatitis
Dermovate Ointment^®^	TaroPharma, Canada	Clobetasol propionate	Mineral Oil; Petrolatum; Lanolin Alcohols	corticosteroid for severe psoriasis, eczema, lichen planus
Nayyar Ointment^®^	Skin Horizons, Jordan	Flaxseed oil, Natural honey, propolis	Petroleum Jelly (Petrolatum), Beeswax (Cera Alba), Linseed (Linum Usitatissimum) Seed Oil, Sweet Almond (Prunus Amygdalus Dulcis) Oil, Propolis Extract, Alkanet (Alkanna Tinctoria) Root Extract	Burn and wound healing, and diabetic foot
Soft Paraffin	Sana Pharma, Jordan	White Soft Paraffin	(Pure) White Soft Paraffin	Emollient, skin protectant

**Table A2:** Rheological parameters corresponding to the Herschel–Bulkley model.

Formulation	Yield Stress Y_2_ (Pa)	Consistency Index (K-Pa·s)	Flow Index (n)
Barrad gel^®^	31.65	78.23	0.30
Doloraz gel^®^	45.46	30.75	0.44
PremaRil AR gel^®^	31.15	34.55	0.39
Betaval cream^®^	10.86	16.35	0.60
Clipp cream^®^	13.14	23.83	0.58
Harrar cream^®^	7.81	59.99	0.31
Betaval ointment^®^	36.23	48.03	0.72
Dermovate ointment^®^	21.72	62.77	0.66
Nayyar ointment^®^	32.17	66.99	0.35
Soft paraffin	46.70	25.76	0.74

**Table A3:** Parameters obtained from the texture analyzer.

Formulation	Hardness (g)	Hardness Work (mJ)	Adhesive Force (g)	Adhesiveness (mJ)
Barrad gel^®^	468.00 ± 42.43	17.95 ± 0.35	216.00 ± 36.77	12.55 ± 2.05
Doloraz gel^®^	318.00 ± 2.83	13.10 ± 0.42	183.00 ± 1.41	8.60 ± 0.28
PremaRil AR gel^®^	334.00 ± 144.25	12.70 ± 1.70	126.00 ± 28.28	7.50 ± 0.00
Betaval cream^®^	309.00 ± 32.53	14.75 ± 0.49	249.00 ± 4.24	9.60 ± 1.84
Clipp cream^®^	429.00 ± 26.87	20.55 ± 2.19	310.00 ± 16.97	12.10 ± 0.71
Harrar cream^®^	262.00 ± 36.77	9.95 ± 0.07	175.00 ± 18.38	7.05 ± 0.35
Betaval oint^®^	1203.00 ± 7.07	57.80 ± 1.41	585.00 ± 270.11	36.35 ± 12.52
Dermovate oint^®^	841.00 ± 66.47	39.15 ± 3.89	529.00 ± 77.78	29.95 ± 11.95
Nayyar oint^®^	261.00 ± 38.18	10.60 ± 2.55	143.00 ± 9.90	6.50 ± 1.41
Soft paraffin	1314.00 ± 48.08	63.00 ± 3.39	725.00 ± 60.81	25.65 ± 2.33

**Table A4:** Post hoc repeated ANOVA pairwise comparisons between yield stress obtained from flow curve, amplitude sweep, and parallel-plate calculations.

(I) Factor1	(J) Factor1	Sig. ^a^	95% Confidence Interval for Difference ^a^	
			Lower Bound	Upper Bound
Flow Curve (Y_2_)	Amplitude Sweep (Y_3_)	0.078	−2.744	56.069
	Parallel-Plate	0.775	−13.742	32.948
Amplitude Sweep (Y_3_)	Flow Curve (Y_2_)	0.078	−56.069	2.744
	Parallel-Plate (Y_1_)	0.004	−28.046	−6.073
Parallel-Plate (Y_1_)	Flow Curve (Y_2_)	0.775	−32.948	13.742
	Amplitude Sweep (Y_3_)	0.004	6.073	28.046

^a^ Significance values (Sig.) and confidence intervals were adjusted using the Bonferroni correction for multiple pairwise comparisons. This conservative adjustment reduces the risk of Type I error by accounting for the number of repeated tests performed. As a result, only differences that remain significant after Bonferroni adjustment can be interpreted as statistically reliable across the comparisons.

**Table A5:** Relative spreadability in different methods.

**Formulation**	**Flow Curve**	Amplitude Sweep	Parallel-Plate cm^2^	Parallel-Plate 1/Pa	Slip-and-Drag	Texture Analyzer	Frictiometer
Barrad gel^®^	1.48	1.32	1.04	1.69	1.03	3.51	0.76
Doloraz gel^®^	1.03	1.35	0.98	1.71	2.20	4.81	0.56
PremaRil AR gel^®^	1.50	2.48	1.14	2.42	1.66	4.96	0.88
Betaval cream^®^	4.30	4.14	1.64	4.86	3.53	4.27	1.67
Clipp cream^®^	3.55	2.04	1.52	3.40	2.01	3.07	1.73
Harrar cream^®^	5.98	7.05	1.34	4.09	2.59	6.33	1.20
Betaval ointment^®^	1.29	1.19	0.85	0.63	0.82	1.09	0.81
Dermovate ointment^®^	2.15	1.46	0.98	0.80	0.59	1.61	0.99
Nayyar ointment^®^	1.46	1.77	1.04	1.57	1.02	5.94	1.81
Soft paraffin	1.00	1.00	1.00	1.00	1.00	1.00	1.00
$Relative\;spreadability=\frac{Spreadability\;Parameter\;of\;formulation}{Spreadability\;Parameter\;of\;soft\;paraffin}\;$							

Spreadability parameter: S(_1–4_) in parallel-plate and slip-and-drag, reciprocal of Y(_1–3_) in parallel-plate (Equation (4)) and rhinometry (flow curve and amplitude sweep), reciprocal of hardness work performed in texture analyzer, and frictiometer ratio in frictiometer.

**Table A6:** Post hoc Friedman’s test pairwise comparisons between the spreadability measurement methods.

Sample 1–Sample 2	Test Statistic	Std. Error	Std. Test Statistic	Sig.	Adj. Sig. ^a^
Parallel-plate (cm^2^)–flow curve stress yield	2.600	0.966	2.691	0.007	0.149
Frictiometer–flow curve stress yield	2.600	0.966	2.691	0.007	0.149
Parallel-plate (cm^2^)–amplitude sweep yield stress	2.500	0.966	2.588	0.010	0.203
Frictiometer–amplitude sweep yield stress	2.500	0.966	2.588	0.010	0.203
Parallel-plate (cm^2^)–frictiometer	0.000	0.966	0.000	1.000	1.000
Parallel-plate (cm^2^)–slip-and-drag	−0.600	0.966	−0.621	0.535	1.000
Parallel-plate (cm^2^)–parallel-plate yield stress Pa	−2.000	0.966	−2.070	0.038	0.807
Parallel-plate (cm^2^)–texture analyzer	−3.500	0.966	−3.623	0.000	0.006
Frictiometer–slip-and-drag	0.600	0.966	0.621	0.535	1.000
Frictiometer–parallel-plate yield stress Pa	−2.000	0.966	−2.070	0.038	0.807
Frictiometer–texture analyzer	3.500	0.966	3.623	0.000	0.006
Slip-and-drag–parallel-plate yield stress Pa	−1.400	0.966	−1.449	0.147	1.000
Slip-and-drag-amplitude sweep yield stress	1.900	0.966	1.967	0.049	1.000
Slip-and-drag–flow curve stress yield	2.000	0.966	2.070	0.038	0.807
Slip-and-drag–texture analyzer	−2.900	0.966	−3.002	0.003	0.056
Parallel-plate yield stress Pa–amplitude Sweep yield stress	0.500	0.966	0.518	0.605	1.000
Parallel-plate yield stress Pa–flow curve stress yield	0.600	0.966	0.621	0.535	1.000
Parallel-plate yield stress Pa–texture analyzer	1.500	0.966	1.553	0.121	1.000
Amplitude sweep yield stress–flow curve stress yield	0.100	0.966	0.104	0.918	1.000
Amplitude sweep yield stress–texture analyzer	−1.000	0.966	−1.035	0.301	1.000
Flow curve stress yield–texture analyzer	−0.900	0.966	−0.932	0.352	1.000

Each row tests the null hypothesis that the Sample 1 and Sample 2 distributions are the same. Asymptotic significances (2-sided tests) are displayed. The significance level is 0.05. ^a^. Significance values have been adjusted by the Bonferroni correction for multiple tests.
