# Supplementary figures and images for: Comparative Evaluation of Spreadability Measurement Methods for Topical Semisolid Formulations/A Scoping Review
Source: Gels. 2025 Dec 12;11(12):1006. doi: 10.3390/gels11121006 (PMC12732428; doi:10.3390/gels11121006)

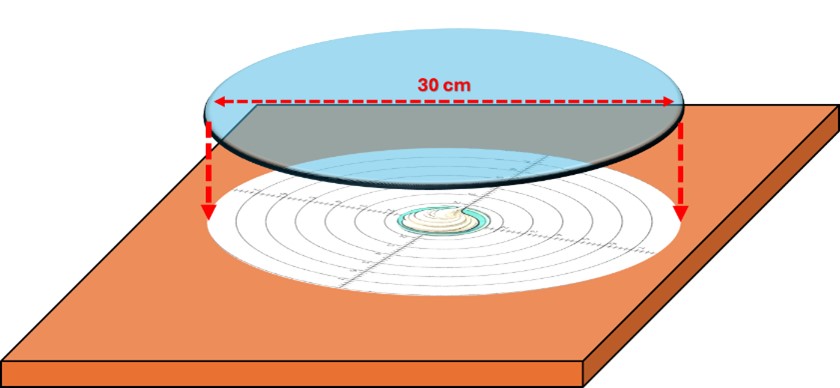

Supplement: Supplementary file 1 [file gels-11-01006-s001.zip › Supplementary Figure S1.jpg]

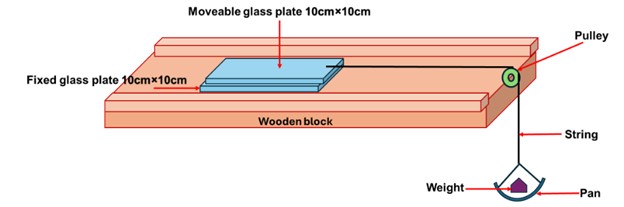

Supplement: Supplementary file 1 [file gels-11-01006-s001.zip › Supplementary Figure S2.jpg]

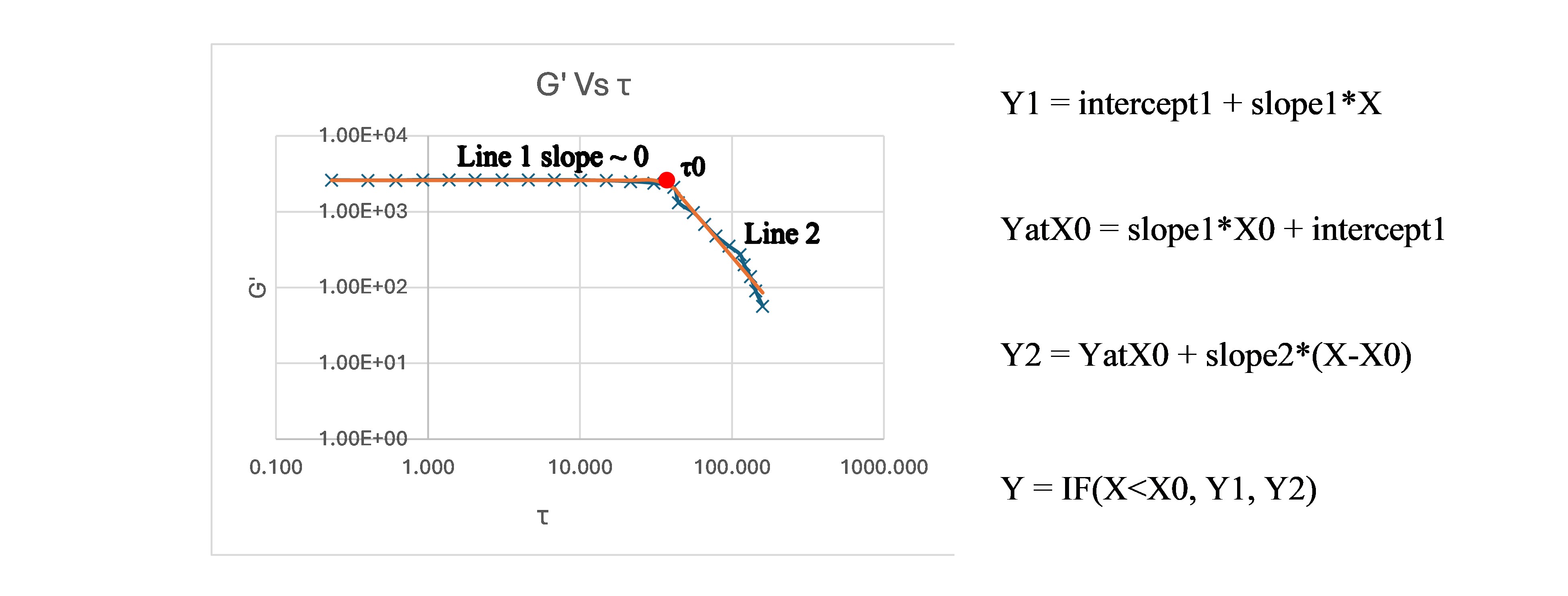

Supplement: Supplementary file 1 [file gels-11-01006-s001.zip › Supplementary Figure S3.jpg]

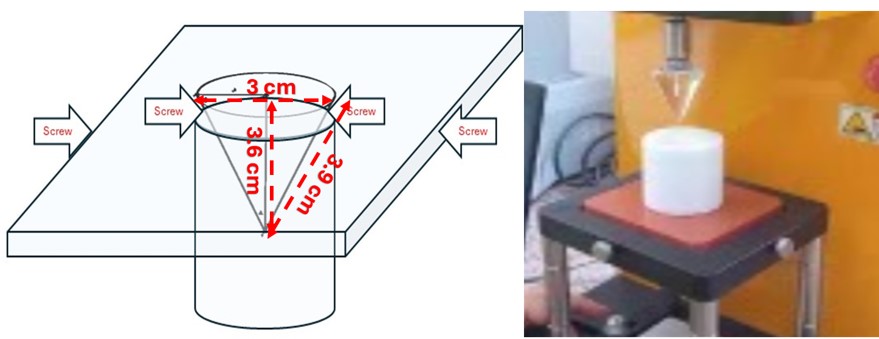

Supplement: Supplementary file 1 [file gels-11-01006-s001.zip › Supplementary Figure S4.jpg]

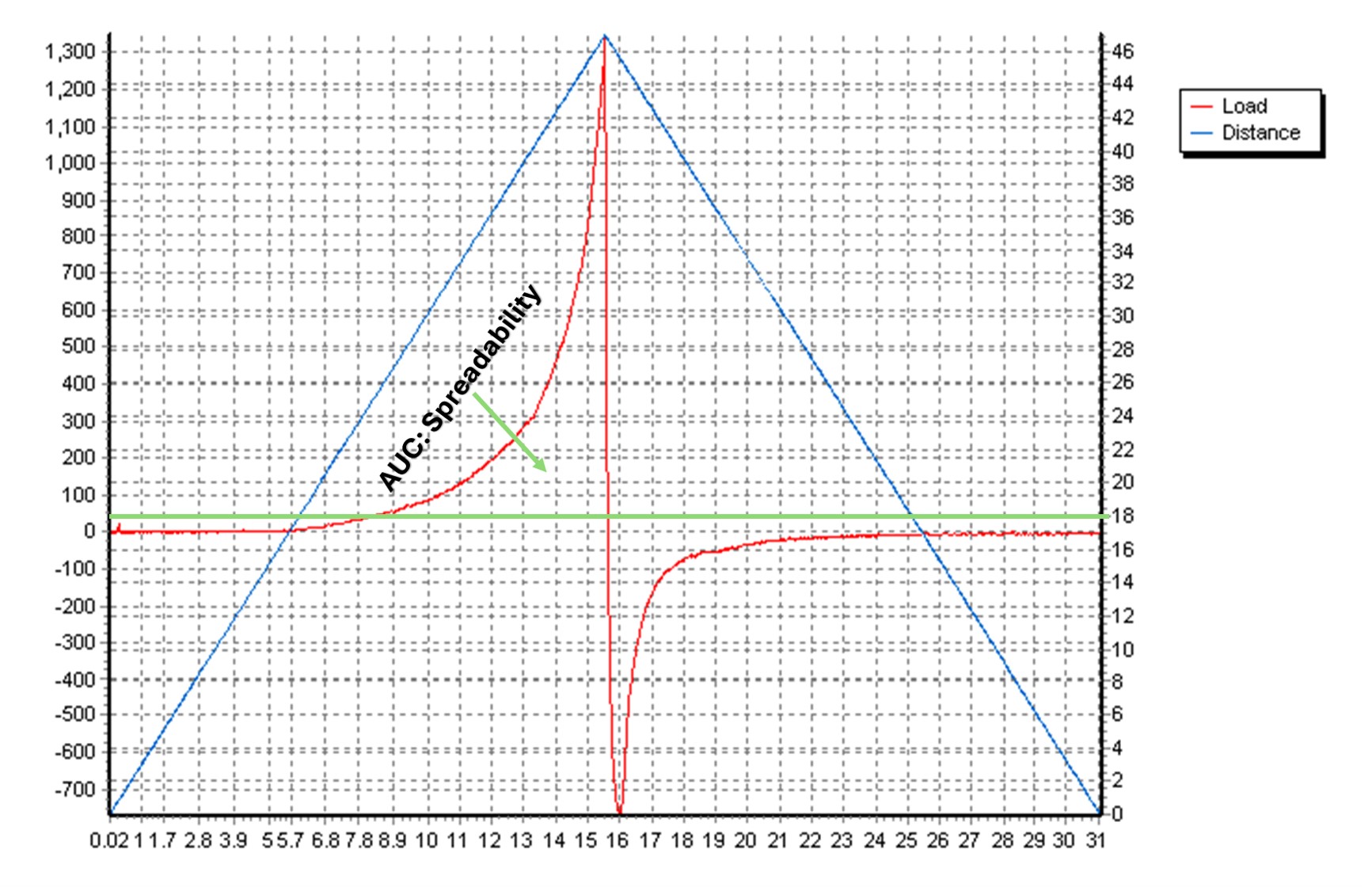

Supplement: Supplementary file 1 [file gels-11-01006-s001.zip › Supplementary Figure S5.jpg]
